# Comparative Genomics of Fermented Vegetable-Derived *Leuconostoc mesenteroides* from Biodiversity Hotspot Yunnan, China

**DOI:** 10.3390/microorganisms14061350

**Published:** 2026-06-16

**Authors:** Yijin Zhu, Haoran Yang, Rong Tang, Sijia Duan, Junfei Chen, Yingli Cai, Ling Zou, Xing Wan, Qiao Shi

**Affiliations:** 1 Agro-Product Processing Research Institute, Yunnan Academy of Agricultural Sciences, Kunming 650221, China; yijin.zhu@outlook.com (Y.Z.);; 2 Institute of Flower Research, Yunnan Academy of Agricultural Sciences, Kunming 650221, China; 3 Department of Microbiology, Faculty of Agriculture and Forestry, University of Helsinki, 00790 Helsinki, Finland

**Keywords:** *Leuconostoc mesenteroides*, pangenome analysis, safety, adaptability, probiotic

## Abstract

Fermented vegetables in Yunnan Province, China, harbor abundant microbial diversity. However, the development of indigenous starter cultures remains under-utilized. Genomic information regarding *Leuconostoc* (*L.*) *mesenteroides* isolates from this region is particularly scarce. To assess the genomic characteristics of eight *L. mesenteroides* isolates from traditional Yunnan fermented vegetables, we performed whole-genome sequencing and conducted a comparative analysis with 21 publicly available vegetable-derived genomes. Comparative genomic analysis revealed marked variation in genome size and plasmid content, and pangenome analysis indicated an open configuration. Core-genome multilocus sequence typing (cgMLST) of the eight indigenous isolates showed high allelic diversity, indicating a genetically heterogeneous and non-clonal population. Phylogenomic analysis revealed that the evolutionary relationships among the 29 strains were not strictly correlated with their vegetable sources, suggesting an influence from other factors, such as geographic origin and region-specific processing methods. Similar to the profiles of the 21 publicly available genomes, inactive prophages, intrinsic vancomycin resistance genes, and genomic island fragments were detected in eight isolates, whereas no known virulence genes were identified. Bacteriocin gene clusters varied among strains, while stress tolerance and probiotic-related genes were conserved. Overall, these results provide genomic indications relevant to the safety, adaptability, and fermentation potential of indigenous *L. mesenteroides* from Yunnan. However, because these functional traits are inferred solely from genomic predictions, subsequent experimental validation is essential to confirm their phenotypic properties and technological efficacy.

## 1. Introduction

Yunnan Province, situated in southwestern China, encompasses a complex topography with diverse climate types, fostering distinctive microbial habitats [1,2]. This environmental variety, coupled with the rich cultural heritage of its numerous ethnic groups, has led to a vast array of traditionally fermented foods. The biological activities from microorganisms play a crucial role in food fermentation, producing various flavor-active and bioactive compounds that contribute to the sensory profiles and nutritional benefits of fermented foods [3,4]. Among lactic acid bacterium (LAB), *Leuconostoc mesenteroides* is found to be the dominant species in many fermented foods, especially in fermented vegetable products [2]. It contributes substantially to initiating the fermentation and shaping the flavor profile of fermented vegetables [5]. Furthermore, *L. mesenteroides* has been demonstrated to possess probiotic attributes and to reduce cholesterol levels and offer protection against hyperlipidemia [6,7]. Moreover, the exopolysaccharides (EPS) generated by *L. mesenteroides* exhibit many advantageous features, including anti-inflammatory, antibacterial, and immunomodulatory effects [8,9]. Taken together, it is of significance to understand *L. mesenteroides* as a fermentation starter in greater genomic details. Notably, vegetable-derived *Leuconostoc* strains have evolved specific adaptations, such as enhanced tolerance to phenolic compounds and oxidative stress [5], along with efficient plant carbohydrate utilization systems [10], which provide competitive advantages in fermenting polyphenol-rich plant matrices like tea, coffee, and legume matrices. Therefore, developing *L. mesenteroides* starters holds significant potential for advancing fermented food production.

Despite the rich diversity of traditional fermented vegetables in Yunnan, the industrial application of indigenous starter cultures from this region remains limited, including those of *L. mesenteroides* [1,2]. Moreover, genomic information on vegetable-derived *L. mesenteroides* isolates from this region is still scarce. Comparative genomic approaches provide valuable tools to investigate genetic diversity, evolutionary relationships, and functional potential among bacterial strains. With the aim of further excavating the biological resources in this region, we isolated eight *L. mesenteroides* strains from traditional fermented vegetables at different locations and sequenced their whole genomes. At the time of analysis, although more than 400 L. mesenteroides genomes were available in the NCBI database, only 21 genomes derived from fermented vegetable-associated sources met the criteria of clear ecological origin and sufficient assembly quality. Therefore, these 21 publicly available genomes were selected for comparative analysis to ensure ecological relevance and data reliability. By conducting a comparative genomic analysis of our eight local isolates and the 21 publicly available genomes, we aimed to explore their genomic diversity, elucidate their phylogenetic relationships, and predict their safety (prophages, genomic islands, antibiotic resistance and virulence determinants), starter potential (bacteriocin loci, stress tolerance-associated genes), and probiotic potential (probiotic-associated genes). These results can serve as an important reference for exploring intraspecies diversity within this taxonomically significant group and for the subsequent selection of indigenous strains as starters for local fermented food industries.

## 2. Materials and Methods

### 2.1. Bacterial Source and Culture Conditions

The eight *L. mesenteroides* strains used in this study are listed in Table 1. They were all isolated from classical naturally fermented vegetables produced using various traditional practices across diverse regions of Yunnan Province, China, and are preserved at the Food Microbial Culture Collection of the Yunnan Academy of Agricultural Sciences (Kunming, China). These eight isolates were activated in the Man-Rogosa-Sharpe (MRS) medium (Hopebio, China). All strains were inoculated in MRS liquid medium and cultivated at 37 °C for 24 h, and the operation was repeated twice to DNA extraction Genomic DNA extraction and whole-genome sequencing were performed by Beijing Biomarker Technologies Co., Ltd. (Beijing, China) following the standard PacBio sequencing protocol, including DNA quality assessment, library construction, library quality control, and sequencing.

### 2.2. Genome Sequencing and Assembly

The genome sequencing, assembly, and annotation results were processed by Beijing Biomarker Technologies Co., Ltd., Beijing, China. The genomes of the eight *L. mesenteroides* isolates were sequenced using HiFi mode on the PacBio platform. For genome assembly, the filtered subreads were assembled using Hifiasm software v0.12-r304, and Circlator v1.5.5 software was utilized for circularisation and adjustment of the starting site. Additionally, Pilon v1.22 software and second-generation data were applied for further error correction to ensure a higher accuracy genome for subsequent analysis. These eight genomes of *L. mesenteroides* isolates were uploaded to GenBank, and the accession numbers are presented in Table 1. The assembly quality metrics for these eight new genomes were presented in Supplementary Table S1.

### 2.3. Pangenome and Phylogenetic Analysis

To investigate the phylogenetic relationships between our isolates and other *L. mesenteroides* strains from similar niches, we performed phylogenetic reconstruction using eight newly sequenced genomes alongside all 21 publicly available vegetable-derived *L. mesenteroides* genomes retrieved from the NCBI genome database (dataset last accessed 27 December 2024; Table 2). Publicly available genomes were selected using a stepwise strategy. First, priority was given to strains clearly annotated as originating from fermented vegetables to ensure ecological relevance. Second, genomes with complete assemblies were preferentially included. When complete genomes were not available, high-quality draft genomes were considered. Because mixing complete and draft assemblies can artificially inflate accessory and cloud gene counts due to gene fragmentation at contig boundaries, strict quality controls were applied to all draft genomes. Specifically, draft assemblies were only included if their genome sizes fell within the expected range for *L. mesenteroides* (approximately 1.8–2.3 Mb). In addition, assembly statistics—such as a low contig number and high annotation completeness—were rigorously evaluated to exclude highly fragmented or aberrant assemblies, thereby ensuring dataset reliability and minimizing assembly-driven bias in the pangenome analysis. The analysis of core and pangenomes, as well as the presence or absence of genes across all 29 *L. mesenteroides* strains, was determined using the Roary pipeline version 3.13.0 [11]. To ensure consistency in gene prediction, all genomes were uniformly annotated using Prokka v1.14.6 [12]. The pangenome accumulation curve was fitted to Heaps’ Law ($n=\kappa {Ν}^{\gamma }$) using R v4.3.2. Visualizations were generated using roary_plots.py. Furthermore, the 29 GFF3 annotations were utilized by the Roary pipeline to calculate the pangenome of the dataset and generate a multiple sequence alignment (MSA) of the core gene (present in more than 99% of strains) using MAFFT and then trim the result using the TrimAI tool at default settings in Phylosuite (v1.2.3). The trimmed alignment was used to infer a maximum-likelihood phylogeny with IQ-TREE v2.3.6, and trees were visualized using the iTOL web tool (https://itol.embl.de/accessed on 15 August 2025 ) [13]. Further strain typing of the *L. mesenteroides* isolates was performed using a core genome multilocus sequence typing (cgMLST) approach. An ad hoc cgMLST schema was constructed *de novo* specifically for this study using chewBBACA (https://github.com/B-UMMI/chewBBACA accessed on 8 December 2025). Allele calling was performed using default parameters, specifically employing a BLAST v2.5.0 Score Ratio (BSR) threshold of 0.6. To define a strict core genome, only loci present in 100% of the analyzed genomes were retained, resulting in a final schema comprising 1556 core loci. Minimum spanning trees (MST) based on the allelic profiles were constructed and visualized using GrapeTree (https://achtman-lab.github.io/GrapeTree/MSTree_holder.html accessed on 8 December 2025). During tree construction, uncalled alleles were handled by pairwise deletion to prevent artificial inflation of allelic distances. Additionally, we conducted an Average Nucleotide Identity (ANI) analysis to assess genomic relatedness. ANI measures the mean identity of homologous genomic regions between two genomes [14]. When two strains have an ANI value of ≥95%, indicating that they are genetically related, they are considered members of the same species [15]. ANI values were computed using FastANI v1.34 and were visualized using the ChiPlot web tool (https://www.chiplot.online/ accessed on 15 August 2025).

### 2.4. Genome Annotation

For additional functional annotations, the core orthogroups shared between 29 strains were analyzed by using KofamKOALA and eggNOG-mapper annotation databases [16]. To screen for bacteriocin operons, we employed the BAGEL 4.0 online prediction tool to annotate the 29 *L. mesenteroides* genomes and investigate the presence of bacteriocin-encoding genes [17]. Virulence factors were predicted using VirulenceFinder 2.03 (https://cge.food.dtu.dk/services/VirulenceFinder/ accessed on 15 August 2025). It should be noted that this database is primarily designed for the identification of virulence-associated genes in well-characterized pathogenic bacteria. Therefore, its coverage of LAB, including *L. mesenteroides*, may be limited. Antibiotic resistance genes were identified using the Resistance Gene Identifier (RGI) tool (https://card.mcmaster.ca/analyze/rgi accessed on 15 August 2025) based on the Comprehensive Antibiotic Resistance Database (CARD). Prophage sequences were identified utilizing PHASTER (https://phaster.ca/ accessed on 17 August 2025) [18]. The IslandViewer 4 tool, accessible at http://www.pathogenomics.sfu.ca/islandviewer/ (accessed on 18 August 2025), was employed for genomic island prediction [19].

## 3. Results and Discussion

*L. mesenteroides* is extensively involved in the sector of vegetable fermentation [20]. Considerable disparities exist across different strains of this species, and the factors contributing to these discrepancies are complex, including the vegetable source, its processing techniques, and geographical positioning, among others. These genomic differences directly influence the strains’ potential for diverse applications. To evaluate the application potential of Yunnan indigenous strains, we sequenced and assembled eight indigenous isolates from traditionally fermented vegetables in Yunnan, China (including *Toona sinensis*, *Brassica juncea*, and *Thlaspi arvense* L.). This is followed by comparative genomic analyses with 21 publicly available vegetable-derived strains retrieved from the NCBI database. Our comprehensive investigation of 29 *L. mesenteroides* genomes elucidated origin-specific genomic characteristics, genetic diversity, and functional attributes, providing critical genomic evidence to support the industrial utilization of these Yunnan isolates.

### 3.1. General Genomic Features of the Eight L. Mesenteroides Isolated Strains

Eight strains of *L. mesenteroides* isolates from fermented vegetables in Yunnan were sequenced using the PacBio platform. The resulting assemblies were complete, with no gaps. The general features of their genomes and the GenBank accession number are displayed in Table 1. For the non-protein-coding genes of the eight strains, there were 12 rRNAs and 71 tRNAs, respectively. All eight strains possess around 2000 predicted protein-coding genes. Among the eight indigenous isolates, XM4 exhibited a relatively large genome (2.10 Mb), while the other seven strains showed similar genome sizes, ranging from 1.97 to 2.05 Mb (Table 1). The genome sizes of the other 21 publicly available vegetable-derived *L. mesenteroides* strains ranged from 1.8–2.23 Mb, with GC content ranging from 37.5% to 38% (Table 2). The variations in genome size may reflect underlying ecological adaptations among the strains [21]. Notably, all isolates harbored plasmids: C5, XM27, XS14, and XS804 each contained a single plasmid (designated pC5-1, pXM27-1, pXS14-1, and pXS804-1, respectively), whereas AP7 and XM4 carried two plasmids, and XS806 possessed three (Table 1). These indigenous *L. mesenteroides* isolates have notable strain-specific variation in the number of plasmids. Functional annotation of these plasmids revealed the presence of genes associated with stress tolerance and environmental detoxification (Supplementary Table S2). Specifically, we identified genes encoding cadmium-transporting ATPase (*cadA*), arsenate reductase (*arsC*), and multicopper oxidase (*mco*), which collectively suggest a specialized mechanism for heavy metal resistance. Additionally, the discovery of thioredoxin systems (*trxA/B*) and glutathione amide reductase (*garB*) indicates the capacity for maintaining redox homeostasis. Such plasmid-borne traits likely provide competitive advantages, enhancing strain survival and fitness under the challenging conditions of fermentation [22].

### 3.2. Average Nucleotide Identity

**Figure 1:**
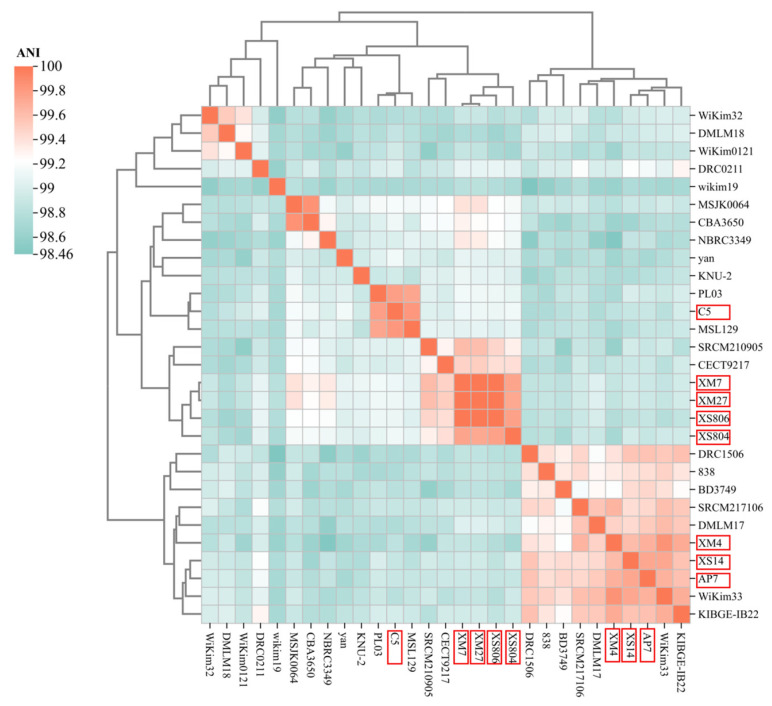
Average Nucleotide identity among all 29 *L. mesenteroides* strains. The isolated eight indigenous strains are circled in red.

Average Nucleotide Identity (ANI) provides a quantitative measure of genomic similarity between microbial strains. As shown in Figure 1, all 29 strains shared ANI values exceeding 98%. Notably, Yunnan isolates of XM7, XM27, XS804 and XS806 shared relatively higher ANI values between each other, while XM4, AP7 and XS14 also exhibit higher value. Additionally, the indigenous strain C5 exhibited high genomic similarity with both MSL129 and PL03.

### 3.3. Pangenome Analysis

**Figure 2:**
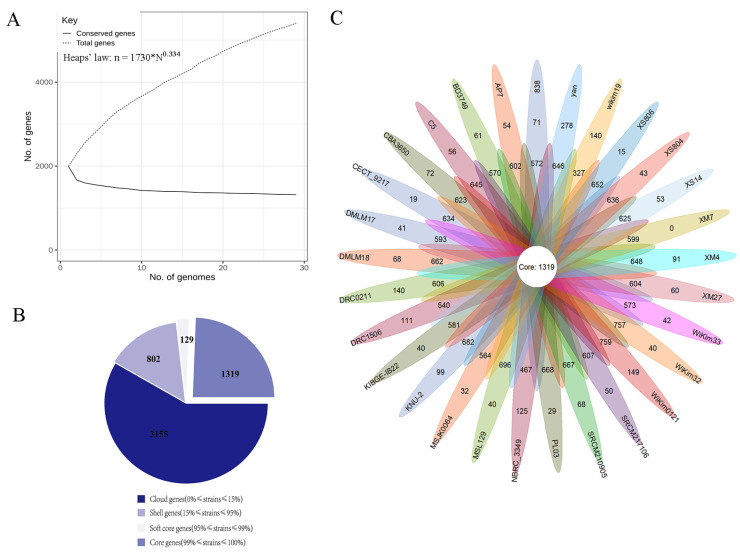
Pangenome characteristics of the 29 *L. mesenteroides* strains. (**A**) Correlation of conserved and total gene counts with the number of genomes, Heaps’ law (n = κN^γ^, γ = 0.334, the asterisk (*) denotes the multiplication sign); (**B**) Composition of the pangenome, showing the percentage distribution of gene categories (core, soft-core, shell, and cloud) across 29 strains; (**C**) Hierarchical “flower plot” visualization of the pangenome structure, with the core genes in the center, accessory genes in the middle ring, and unique genes in the outer ring.

Roary analysis revealed that the pangenome of the 29 *L. mesenteroides* comprises 5405 genes. Among these, 1319 genes (24.4%) constituted the core genome (present in all 29 strains), while 129 genes (2.4%) formed the soft-core genome (present in 95–99% of strains). The sum of core and soft-core genomes, encompassing 1448 genes, accounted for 26.8% of the total pangenome. The accessory genome was composed of 802 shell genes (14.8%) (present in 15–95% of strains) and 3155 cloud genes (58.4%) (present in fewer than 15% of strains) (Figure 2B). It should be noted that differences in genome assembly quality (e.g., complete versus draft assemblies) may influence the estimation of accessory and cloud gene content. Specifically, gene fragmentation at contig boundaries or incomplete gene prediction in draft genomes can lead to an overestimation of accessory gene categories. Although strict inclusion criteria were applied in this study to ensure high and comparable assembly quality and minimize such bias, minor deviations may still exist. Therefore, the observed pangenome patterns should be interpreted with caution, with emphasis placed on overall trends rather than absolute gene counts. The core genes, shared by all strains, represent conserved genomic elements that may support common phenotypic traits fundamental to the species. As shown in Figure 2A, the core gene count stabilized with increasing sample size, while the numbers of accessory and unique genes exhibited a corresponding increase. The pangenome expansion was modeled using Heaps’ law, which yielded a growth exponent γ of 0.334, suggesting that the *L. mesenteroides* pangenome possesses open characteristics within the sampled population. Analysis of the core, accessory, and unique gene content revealed substantial variation among the 29 strains (Figure 2C). The proportion of core genes relative to the total gene count in each strain ranged from 58.81% (Yan) to 73.85% (WiKim19). Among the eight Yunnan isolates, XM4 exhibited a relatively low core gene content (64.09%), while XM7 showed a higher proportion (68.77%); the remaining isolates displayed similar core gene percentages (around 66%). Accessory gene proportions varied from 18.31% (WiKim19) to 35.78% (WiKim32). The proportions of unique genes varied considerably among the strains, indicating the genetic diversity among them. Particularly, XM7 and XS806 contained the lowest percentages of unique genes among all strains, with XM7 possessing none, which suggests their genetic makeup is highly conserved within the population. In contrast, strain Yan possessed a significantly higher proportion of unique genes compared to all other isolates. Among the local isolates, XM4 had the highest proportion of unique genes, suggesting potential functional diversification and possible ecological relevance [23]; however, this hypothesis requires further functional validation.

### 3.4. Phylogeny of the Core Genome

**Figure 3:**
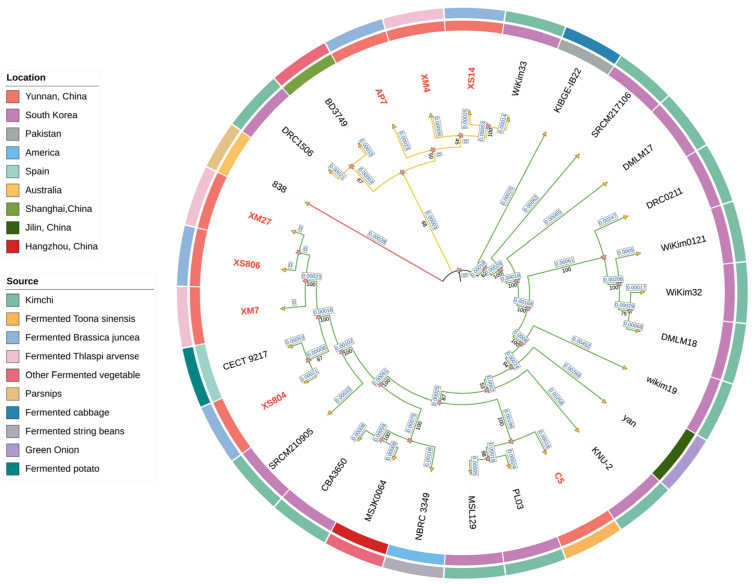
Maximum-likelihood phylogenetic tree showing the relationships of eight local strains (highlighted in red) among 29 *L. mesenteroides* genomes based on core-genome alignment. The tree is unrooted, as no outgroup was included. Branch lengths represent genetic distances inferred from the core genome. Values shown in blue boxes along branches indicate branch lengths. Numbers at the nodes represent bootstrap support values. The stars indicate the internal nodes of the phylogenetic tree, representing the common ancestors of the respective clades. The arrows serve as navigational aids, pointing from the tips of the phylogenetic branches to their corresponding strain. The outer ring indicates the source of isolation, and the inner ring represents the geographic origin of each strain.

A phylogenetic tree of the 29 *L. mesenteroides* core genomes was reconstructed using maximum likelihood analysis (Figure 3). The resulting phylogeny suggests a possible association with both vegetable source and other factors, such as geographic origin and regional-specific fermentation practices. Several indigenous strains (XM7, XM27, XS806) from two vegetable sources formed a distinct cluster, indicating close genetic relatedness among these isolates with local processing methods. Additionally, XM7 and XM27 (both isolated from fermented *Thlaspi arvense*) exhibited close phylogenetic relatedness, whereas XM4 (also from fermented *Thlaspi arvense*) was positioned on a separate branch, clustering with XS14 (from fermented *Brassica juncea*) and a Korean kimchi isolate, highlighting that factors beyond vegetable source—such as strain-specific evolutionary histories or localized fermentation practices—may contribute to the observed phylogenetic patterns. Strain C5, isolated from traditional fermented *Toona sinensis* in Yunnan, clustered phylogenetically with Korean kimchi isolates. This relatedness may reflect the influence of shared ecological niches associated with plant-based fermentation. Further investigation into functional genes involved in substrate utilization would help elucidate the genetic basis underlying this clustering pattern. The close genetic relationship between C5, PL03, and MSL129 aligns with their high genomic similarity (ANI > 99%), suggesting potential shared functional characteristics. Given that PL03 is a known high-mannitol-producing strain capable of fermenting premium kimchi [24], the closely related C5 strain might possess equivalent fermentation potential, but this requires experimental confirmation. Furthermore, strains AP7, XM4, and XS14 share a common ancestral node with DRC1506, which is renowned for its exceptional acid tolerance and mannitol production capabilities, and probiotic properties [25]. Additionally, AP7 has been demonstrated to possess antimicrobial properties [26]. The shared evolutionary history of this branch implies that XM4 and XS14 may similarly exhibit these desirable acid tolerance and metabolic traits, but direct functional studies are necessary to verify this hypothesis. This phylogenetic framework provides a robust foundation for future functional studies targeting these specific strains.

**Figure 4:**
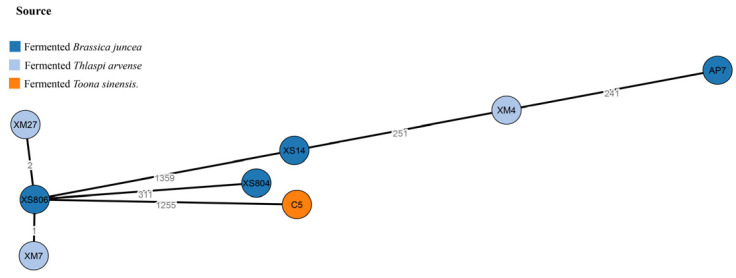
Minimum spanning tree (MST) based on cgMLST analysis of eight *L. mesenteroides* isolates. Numbers on the connection lines represent the allelic difference between isolates. The isolates are colored according to their isolation source.

To obtain more insight into the genetic relationship of the eight *L. mesenteroides* isolates derived from various spontaneously fermented vegetables in Yunnan, a minimum spanning tree (MST) (Figure 4) was generated by the core-genome multilocus sequence typing (cgMLST) analysis. A subset of isolates (XM7, XM27, XS806) clustered tightly with small allelic distances (1–2), indicating close genetic relatedness. In contrast, other isolates (C5, XS804, XM4) occupied long branches with hundreds to over a thousand allelic differences, reflecting greater divergence at the core-genome level. Moreover, clustering does not strictly correlate with the sources of isolation, as strains from the same fermented vegetable (e.g., XM4, XM27 and XM7) fell into different groups, whereas isolates from distinct substrates (e.g., XS14, AP7 and XM4) clustered closely together. The allelic diversity revealed by cgMLST demonstrates that the Yunnan *L. mesenteroides* isolates represent a genetically broad and heterogeneous rather than a homogeneous, clonal population. Similar findings were also observed in *Lactobacillus plantarum*. A multilocus sequence typing (MLST) analysis of 186 *Lactobacillus plantarum* isolates identified substantial intraspecies diversity even among isolates from similar ecological niches [27].

### 3.5. COG and KEGG Analysis of the Core Genome

**Figure 5:**
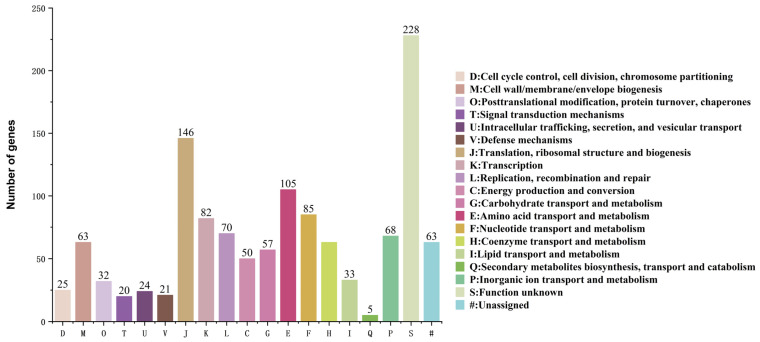
COG classification of the core genome among 29 *L. mesenteroides* strains.

Among the 1319 core genes, 1240 were successfully assigned to 19 Clusters of Orthologous Genes (COG) categories (Figure 5). The largest proportion of genes (18.38%) were categorized into the ‘Function unknown’ group, indicating that a substantial fraction of conserved genes in *L. mesenteroides* remains uncharacterized. The most abundant functional category was ‘Translation, ribosomal structure and biogenesis’ (11.77%). This genetic enrichment is generally associated with the cellular machinery required for growth, metabolic responses, and product synthesis in *L. mesenteroides* [28]. Significant proportions of genes were also classified into ‘Amino acid transport and metabolism’ (8.47%) and ‘Nucleotide transport and metabolism’ (6.85%), which are fundamental to core cellular processes like growth and replication. Amino acid metabolism, in particular, is closely linked to the development of flavor compounds in fermented foods [29,30]. Notably, the quantity of core genes associated with secondary metabolites biosynthesis, transport, and catabolism was low, reflecting the inherently limited secondary metabolic repertoire of LAB.

**Figure 6:**
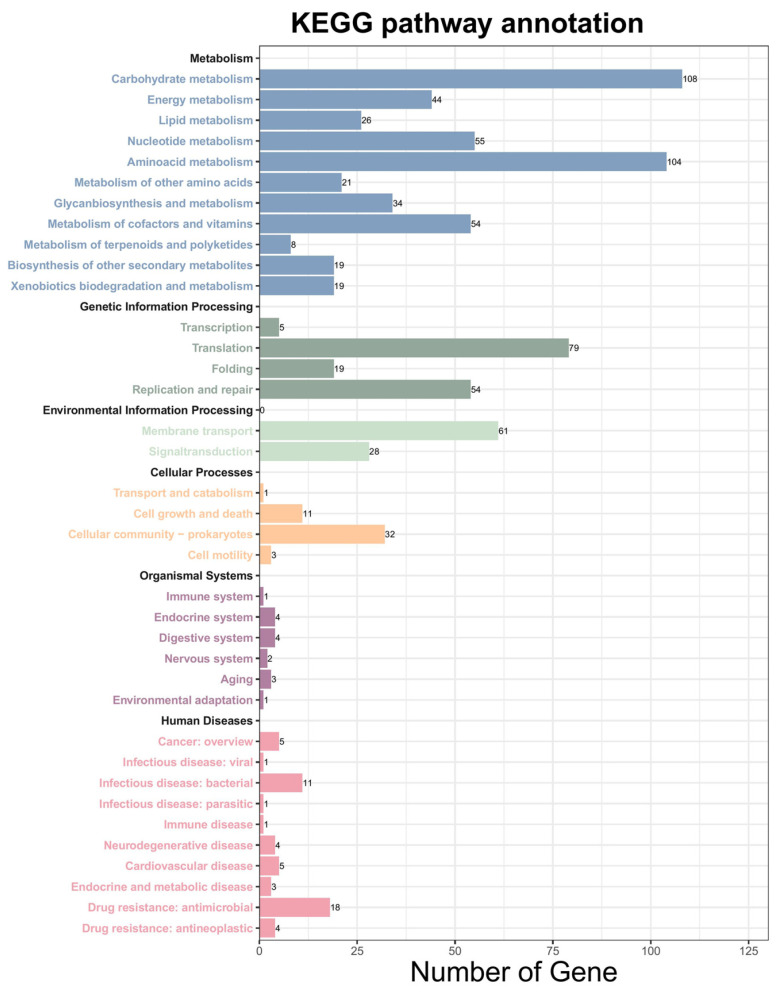
KEGG classification of the core genome among 29 *L. mesenteroides* strains.

Kyoto Encyclopedia of Genes and Genomes (KEGG) pathway analysis (Figure 6) further confirmed that the core genome is heavily involved in core metabolic processes, primarily carbohydrate and amino acid metabolism. Specifically, we identified 108 genes involved in carbohydrate metabolism, 104 in amino acid metabolism, and 55 in nucleotide metabolism. Within genetic information processing, genes were predominantly enriched in translation, replication, and repair. Separately, under environmental information processing, 61 genes were implicated in membrane transport, crucial for substrate uptake, waste excretion, and cellular homeostasis. While 18 genes were annotated with functions related to antimicrobial resistance mechanisms, such as efflux pumps, these annotations do not imply pathogenicity or clinically relevant resistance, but rather reflect the intrinsic resistome involved in basic cellular homeostasis and stress tolerance.

### 3.6. Distribution and Characterization of Prophages and Genomic Islands in L. mesenteroides Genomes

Using PHASTER, prophage regions were predicted in 29 *L. mesenteroides* genomes and classified into intact, questionable, and incomplete categories based on PHASTER’s scoring criteria, which integrate the presence of phage-like CDS, structural/functional phage gene matches, and total region coverage against known phage databases. Intact prophages (>90 score) generally contain a full complement of structural and lifecycle-associated genes and are considered more likely to represent complete prophage elements, whereas questionable (70–90) and incomplete (<70) regions likely represent degraded remnants with fewer phage gene features.

Except for DRC0211 and MSJK0064, all other strains contained 1–4 prophage regions, and most of these genomes harbored at least one high-confidence (intact) prophage alongside questionable or incomplete prophage elements (Supplementary Table S3). High-scoring (typically 120–150) intact prophage regions were identified on the host chromosome, with predicted lengths ranging from ~20.2 kb to ~62.5 kb (except for XM27 at ~102.6 kb). These regions encoded multiple typical phage structural and lifecycle-associated genes, such as lysin, capsid, tail, head, protease, portal, and terminase, indicating the presence of relatively complete phage gene modules consistent with canonical prophages.

In some of the predicted intact prophage regions, genes encoding classical integrase were not detected, which may reflect partial loss of integrase genes during prophage evolution or alternative integration mechanisms. While lack of integrase may impact classical prophage excision/recombination mechanisms, whether it reduces the stability of prophage maintenance or its likelihood of activation in the host genome requires experimental validation. The GC content of the prophage regions predicted by PHASTER was similar to the overall host genome GC content (difference < 5%), suggesting long-term integration of these prophages in the host genomes. Questionable and incomplete prophage regions exhibited lower completeness scores (10–80) and shorter lengths (15.2–49.1 kb). These regions frequently lacked certain structural or functional genes, likely representing prophage remnants that have undergone degradation through gene loss or recombination during host evolution, thereby contributing to genomic variation among strains [31]. Importantly, PHASTER classifications only indicate the similarity and completeness of predicted prophage regions relative to known phage gene features; they do not directly predict whether these prophages will be induced into the lytic cycle under specific environmental stimuli. Previous studies have demonstrated that even incomplete or questionable prophages can sometimes produce inducible phage particles under stress conditions [32]. Beyond their viral functions, prophages often carry ‘cargo genes’ that confer adaptive advantages, actual experimental validation is thus necessary to truly determine whether these strains are safe, stable, and suitable for industrial applications.

Across all 29 strains, we also identified at least two genomic islands (GIs) per genome (Supplementary Table S4). As important components of mobile genetic elements, GIs can encode diverse functions—including metabolic pathways, symbiotic factors, resistance determinants, secretion systems, and pathogenicity factors—depending on the repertoire of genes they carry [33]. Functional annotation of the genes within these genomic islands, performed using IslandViewer 4, revealed no detectable virulence or significant antibiotic resistance genes. However, the absence of such genes in genomic analyses does not constitute conclusive evidence of safety, and further experimental validation is required to assess the safety of these strains.

### 3.7. Bacteriocin-Related Gene Clusters in L. mesenteroides

Bacteriocins are ribosomally synthesized antimicrobial peptides that contribute to food preservation. Compared to chemical preservatives, they are non-toxic and innocuous. To assess the bacteriocin biosynthetic potential of *L. mesenteroides*, all 29 genomes were analyzed using the BAGEL4 online platform. Among these genomes, 25 strains contained one bacteriocin-associated Area of Interest (AOI), whereas no recognizable bacteriocin gene clusters were detected in strains C5, MSL129, PL03, or DRC0211 (Table 3).

Most AOIs were annotated as encoding Enterocin X–related peptides, which belong to class IIb bacteriocins. In all Enterocin X–positive strains, only the gene encoding the Enterocin Xβ precursor peptide was detected, while the corresponding Xα peptide gene was not identified within the AOI or through additional BLAST searches against the entire genome. The absence of the cognate Xα peptide suggests that these strains may harbor an incomplete or non-functional Enterocin X-like locus. Further investigation would be required to confirm any potential antimicrobial activity. The predicted Enterocin Xβ precursor peptides were approximately 56 amino acids in length, consistent with the typical size of class II bacteriocin prepeptides. Furthermore, the majority of Enterocin Xβ–encoding clusters contained adjacent genes annotated as ABC transporters, which are commonly associated with bacteriocin secretion and immunity (Supplementary Table S5). In contrast, strain Yan uniquely harbored a bacteriocin gene cluster annotated as sonorensin. The open reading frame for the synthesis of sonorensin bacteriocin consisted of 208 amino acids, which is markedly larger than the Enterocin Xβ peptides identified in other strains. No ABC transporter gene was identified within the sonorensin cluster. Although no AOI was detected in the genome of C5 via BAGEL4, the presence of highly divergent and uncharacterized antimicrobial peptide genes cannot be excluded. Further analysis would be required to confirm their presence.

Class II bacteriocins are generally small, heat-stable, and non-modified peptides and are subdivided into subclasses IIa–IId based on peptide composition and functional properties. Among these, class IIb bacteriocins typically consist of two complementary peptides that together mediate antimicrobial activity [34,35]. In this study, most *L. mesenteroides* genomes were predicted to encode Enterocin X bacteriocins belonging to class IIb. However, only the Enterocin Xβ was identified at the genomic level. The lack of the cognate Xα peptide makes the functional status of these clusters uncertain. Prior research has also observed *L. mesenteroides* harboring the Enterocin Xβ peptide alone [36], which might be attributed to limitations in the automated annotation of short open reading frames, or a potential non-canonical functional mechanism in *L. mesenteroides* that warrants further manual curation and investigation. And the sonorensin has been reported to exhibit broad-spectrum antimicrobial activity against both Gram-positive and Gram-negative bacteria, highlighting its potential relevance for food-related applications [36].

### 3.8. Antibiotic Resistance and Virulence

To assess the potential health risks of the 29 *L. mesenteroides* strains, we conducted a comprehensive analysis of both antibiotic resistance and virulence genes. Antibiotic resistance genes were predicted using the Comprehensive Antibiotic Resistance Database (CARD) via the Resistance Gene Identifier (RGI) tool. All strains showed low-identity hits to sequences annotated as *vanT* and *vanY*, with sequence identities ranging from 32.35% to 36.65% (Supplementary Table S6). These hits likely represent distantly related homologous sequences rather than confirmed resistance determinants.While intrinsic vancomycin resistanceoften mediated by the alteration of peptidoglycan precursors [37,38,39]—is widely recognized in *Leuconostoc* spp. and other LAB, low-identity genomic hits alone are insufficient to claim active resistance at the strain level. Only strain CECT9217 harbored a complete *aadA* gene cluster (exhibiting 100% identity), which confers resistance to spectinomycin and streptomycin via antibiotic inactivation [40]. Crucially, no virulence-associated genes were identified in any strain using the VirulenceFinder web tool, supporting their safety profile for potential applications.

### 3.9. Genomic Basis of Stress Response and Tolerance in L. mesenteroides

**Figure 7:**
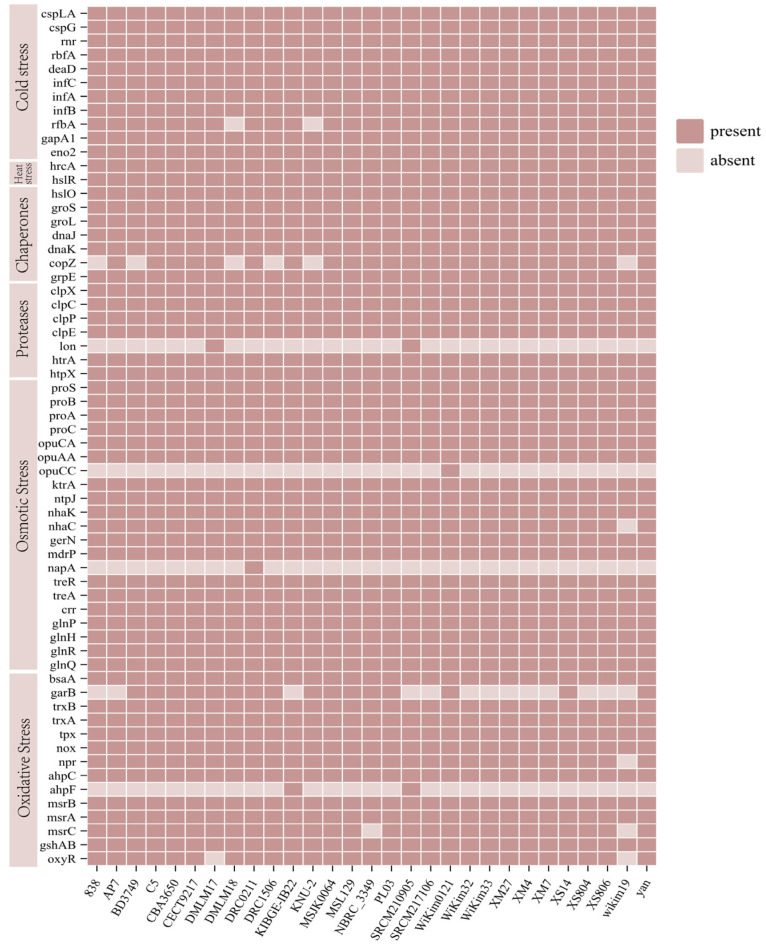
Presence and absence of putative genes related to stress response and tolerance in *L. mesenteroides*.

Comparative genomic analysis of the 29 *L. mesenteroides* strains revealed a diverse repertoire of genes potentially involved in responses to multiple environmental stresses, including cold, heat, osmotic, and oxidative conditions. These genetic features indicate a baseline genomic potential for environmental resilience that may influence strain performance during vegetable fermentation (Figure 7), though phenotypic robustness under actual fermentation conditions remains to be experimentally verified. For cold-stress response, all strains harbored genes encoding cold shock proteins (*cspLA* and *cspG*), together with auxiliary translational regulators, including ribonuclease R (*rnr*), ribosome-binding factor A (*rbfA*), and the RNA helicase (*deaD*). These genes are generally known to be essential for mRNA stabilization and maintaining translational efficiency at low temperatures. Similar cold-stress response gene sets have been reported in other LAB, such as *Lactiplantibacillus plantarum* and *Weissella cibaria*, suggesting a conserved genomic features associated with cold-stress response in these vegetable-derived strains [41,42]. Although these genes are typically upregulated during short-term cold exposure and downregulated during prolonged low temperatures, their identification in all genomes suggests a shared genomic potential for low-temperature adaptation, highlighting the theoretical potential of these strains for applications in vegetable fermentation at low temperatures.

In addition to regulatory factors, genes involved in central carbon metabolism and cell integrity, including glyceraldehyde-3-phosphate dehydrogenase (*gapA1*) and enolase (*eno2*), were also identified. These genes have been implicated in maintaining metabolic flux and cellular structure during cold stress. Collectively, the presence of these determinants suggests that the analyzed strains possess a genetic basis consistent with a genomic potential related to cellular processes under low-temperature conditions (5–15 °C), although transcriptional or phenotypic data would be required to confirm their active role. Indeed, previous studies have confirmed that *L. mesenteroides* plays a pivotal role in driving the fermentation process at such temperatures [43].

Genes associated with heat stress regulation were conserved across all strains, including the transcriptional repressor *hrcA* and the heat shock protease *hslR*. In LAB, HrcA regulates the expression of major chaperone operons, such as *dnaK* and *groEL*, while HslR contributes to protein quality control under elevated temperatures. The presence of these genes suggests that *L. mesenteroides* strains harbor conserved regulatory systems for associated with protein quality control under thermal stress, which are frequently encountered during the exothermic phases of vegetable fermentation [44,45], though their functional activation in these specific strains requires further empirical validation.

All genomes encoded core genetic determinants predicted to be involved in the osmotic stress response, primarily involving ion homeostasis and compatible solute management. The Na^+^/H^+^ antiporter system (*nhaCK*), together with potassium uptake systems, likely contributes to maintaining intracellular ionic balance under hyperosmotic conditions. Although genes involved in endogenous glycine betaine biosynthesis were absent, all strains encoded components of the OpuA ABC transporter system (*opuAA* and *opuCA*), potentially enabling the uptake of exogenous osmoprotectants such as glycine betaine and carnitine. Notably, a complete OpuC transporter system was largely absent; while *opuAA* and *opuCA* were universally present, *opuCC* was uniquely identified in strain WiKim0121. This incomplete transporter repertoire may limit the efficiency of compatible solute uptake, a phenomenon previously observed in other Gram-positive bacteria [46,47]. In contrast, all strains harbored complete proline biosynthesis pathways (*proSBAC*), which may support endogenous accumulation of proline as a major osmoprotectant [48]. Although trehalose-related genes (*treA* and *treR*) were present, the absence of *treB* and *treP* suggests a limited genetic capacity for trehalose utilization [49]. Together, these findings indicated that, based on genomic predictions, *L. mesenteroides* strains may be associated with mechanisms such as proline accumulation and ion homeostasis to cope with hyperosmotic environments typical of fermented foods.

All strains harbored conserved core genetic components associated with oxidative stress defense, including both glutathione- and thioredoxin-dependent redox systems. Genes encoding glutathione peroxidase (*bsaA*) and a bifunctional glutathione synthetase (*gshAB*) were universally present; however, the capacity for complete glutathione biosynthesis remains uncertain, as *gshAB* alone may not suffice for glutathione production in LAB [50]. Additionally, glutathione amide reductase (*garB*) was detected in 16 strains, including indigenous isolates C5 and XS14, although its functional role in LAB remains unclear [51]. All strains encoded a conserved thioredoxin system, including thioredoxin reductase (*trxA*), thioredoxin (*trxB*), thiol peroxidase (*tpx*), and protein repair enzymes (*msrA*, *msrB*, *msrC*). Genes encoding alkyl hydroperoxide reductase subunit C (*ahpC*) were present in all genomes, whereas the complete AhpCF system, including *ahpF*, was identified only in strains KIBGE-IB22 and SRCM210905. The presence of this complete complex suggests that these two strains have the genomic potential for enhanced scavenging capabilities for hydrogen peroxide and organic peroxides, suggesting a potential role in oxidative stress response [52], pending future phenotypic confirmation.

### 3.10. Genomic Basis of Probiotic-Related Traits of L. mesenteroides

**Figure 8:**
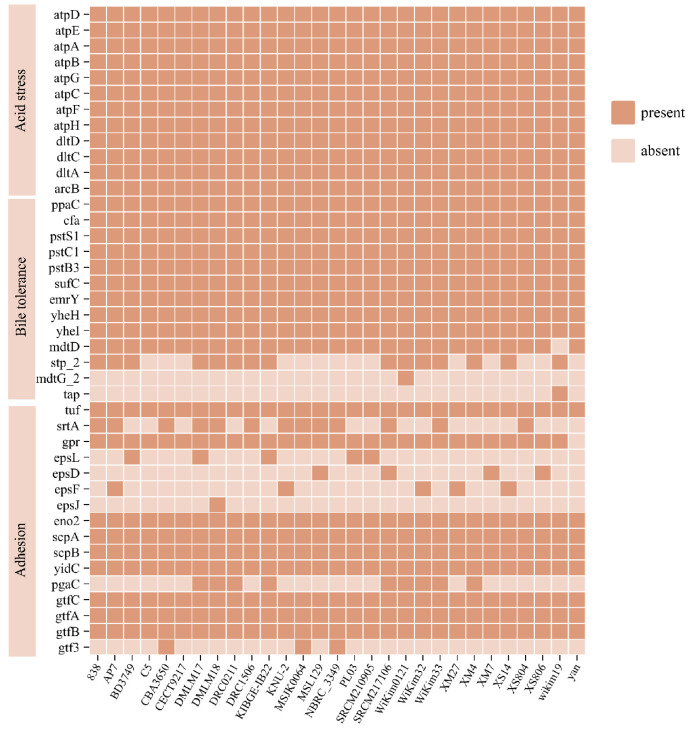
Presence and absence of putative genes related to Probiotic-Related Traits in *L. mesenteroides*.

Acid stress is a common challenge encountered by LAB during fermentation due to the accumulation of organic acids, and during gastrointestinal transit due to exposure to gastric acid. Genomic analysis revealed that all *L. mesenteroides* strains possessed a complete F_1_F_0_-ATPase operon (*atpACDGH* and *atpBEF*) (Figure 8), suggesting a conserved genetic potential for ATP-dependent proton extrusion as a primary acid stress response. However, once the pH value in the external environment reaches 3.5, bacteria would be challenged to maintain the transmembrane potential, leading to the cessation of hydrogen ion efflux and the subsequent inhibition of cellular metabolism [53]. Notably, alternative acid resistance pathways were largely absent in these strains. None of them encoded the glutamate decarboxylase (GAD) system, and the arginine deiminase (ADI) pathway was found to be incomplete. Although the *arcB* gene encoding ornithine transcarbamoylase was identified, key enzymes required for ammonia production and arginine-mediated pH homeostasis, including arginine deiminase (*arcA*) and carbamate kinase (*arcC*), were absent. This incomplete ADI pathway likely limits the contribution of arginine catabolism to intracellular pH regulation.

Cell envelope modification represents another strategy for acid adaptation. Genes encoding components of the *dlt* operon, including *dltA*, *dltC*, and *dltD*, were detected; however, the absence of *dltB* suggests an incomplete D-alanylation process of lipoteichoic acids. This may reduce the ability of these strains to modulate cell surface charge and maintain cell wall stability under acidic conditions. Collectively, these genomic features indicate that *L. mesenteroides* strains rely predominantly on F_1_F_0_-ATPase–mediated proton extrusion for acid stress response, while possessing limited auxiliary acid resistance mechanisms.

Genomic analysis indicated that none of the strains encoded bile salt hydrolase (BSH) genes, indicating a lack of specialized enzymatic mechanisms for direct bile salt modification. While multiple multidrug resistance (MDR) efflux transporters were identified, including *emrY*, *yheH*, *yheI*, *mdtD*, *mdtG_2*, *tap*, and *stp_2*, inferring specific bile resistance from these generalized systems is highly indirect. For instance, although *emrY* has been previously implicated in bile resistance in other bacteria [54], its analogous role in *L. mesenteroides* remains speculative without targeted phenotypic validation. Additional genes associated with general membrane integrity and lipid metabolism, including *ppaC* (manganese-dependent inorganic pyrophosphatase) and *cfa* (Cyclopropane-fatty-acyl-phospholipid synthase), were also conserved across the genomes. These genes have been linked to broad environmental stress responses, which can sometimes include enhanced bile tolerance in other Gram-positive bacteria by stabilizing membrane structure and composition [55,56]. Several efflux transporters exhibited strain-specific distributions; for example, *stp_2* was detected in 15 strains, including indigenous isolates AP7, XM4, and XS14, whereas *mdtG_2* and *tap* were uniquely identified in strains WiKim0121 and WiKim19, respectively. However, the capacity of these generalized transporters to specifically extrude bile salts in *L. mesenteroides* remains unconfirmed.

Genes annotated with potential roles in adhesion and host interaction displayed substantial inter-strain variability. A conserved core set of such genes, including *tuf*, *gpr*, *eno2*, *scpA*, *scpB*, *yidC*, and *gtfCAB*, were identified in all strains. However, it is critical to note that several of these markers (such as *tuf*, *eno2*, and *yidC*) are primarily essential housekeeping or multifunctional proteins. For instance, the *tuf* gene encodes elongation factor Tu (EF-Tu), a protein fundamentally required for protein synthesis across all bacteria; although it has been reported to participate in bacterial adhesion in some LAB [57], its universal genomic presence alone is insufficient to infer actual adhesion capacity. Sortase A (*srtA*), which mediates the anchoring of surface proteins to the cell wall, was present in 13 strains, including AP7 and XS804. In other Gram-positive bacteria, Sortase A has been shown to play a role in epithelial adhesion, theoretically providing a structural basis for potential host interaction in these strains [58]. The *espJ* gene encoding a glycosyltransferase was uniquely identified in strain DMLM18. Genes involved in exopolysaccharide (EPS) biosynthesis and poly-β-1,6-N-acetyl-D-glucosamine (PNAG) production, including *pgaC*, were variably distributed among the strains. Although PNAG has been implicated in biofilm formation and intercellular adhesion in Gram-positive bacteria, the functional impact of these genes on the adhesion of *L. mesenteroides* requires experimental validation. Taken together, these genomic features highlight a theoretical framework for diverse adhesion potentials among *L. mesenteroides* strains, emphasizing the importance of strain-specific evaluation of probiotic-related properties.

## 4. Conclusions

In this study, a comprehensive comparative genomic analysis was performed on eight *L. mesenteroides* strains isolated from traditional fermented vegetables in Yunnan, China, together with 21 publicly available genomes retrieved from the NCBI database. The integrated analysis provides a framework for exploring their phylogenetic relationships, genomic safety features, and genetic determinants potentially associated with stress response, environmental adaptability, and probiotic-related traits. These findings provide a valuable genomic resource for vegetable-derived *L. mesenteroides* and highlight that strains from Yunnan fermented vegetable exhibit notable genetic diversity, despite overall high genomic homology. It is worth noting that the functional roles of the identified genes were inferred solely from in silico predictions and and should be considered hypothesis-generating rather than definitive. Therefore, further phenotypic characterization and targeted selection are necessary to confirm gene functions, which will be essential for fully assessing the technological and probiotic potential of these indigenous strains and facilitating their future application in food production.

## Figures and Tables

**Table 1:** The assembly information of the genomes of eight isolates.

Sample ID	Origin	Source	Accession Number	Size (Mb)	GC Content (%)	Contig Number	Chromosome Size (bp)	Plasmid Number	Plasmids	No. of rRNA	No. of tRNA	Predicted Geneset Number
C5	Yunnan, China	Fermented *Toona sinensis*	JBUEEF000000000	2.05	37.72	2	2,012,980	1	pC5-1:35,370 bp	12	71	2024
AP7	Yunnan, China	Fermented *Brassica juncea*	JBUEEG000000000	2.01	37.7	3	1,948,310	2	pAP7-1:37,366 bp; pAP7-2:20,698 bp	12	71	1979
XM7	Yunnan, China	Fermented *Thlaspi arvense*	JBUEEH000000000	1.97	37.69	2	1,930,160	1	pXM7-1:37,365 bp	12	71	1918
XM4	Yunnan, China	Fermented *Thlaspi arvense*	JBUEEI000000000	2.10	37.56	3	2,036,013	2	pXM4-1:37,372 bp; pXM4-2:22,804 bp	12	71	2062
XM27	Yunnan, China	Fermented *Thlaspi arvense*	JBUEEJ000000000	1.97	37.69	2	1,972,367	1	pXM27-1:37,365 bp	12	71	1983
XS14	Yunnan, China	Fermented *Brassica juncea*	JBUEEK000000000	1.99	37.69	2	1,989,307	1	pXS14-1:37,364 bp	12	71	2001
XS804	Yunnan, China	Fermented *Brassica juncea*	JBUEEL000000000	2.04	37.63	2	2,001,145	1	pXS804-1:37,365 bp	12	71	2002
XS806	Yunnan, China	Fermented *Brassica juncea*	JBUEEM000000000	2.02	37.64	4	1,930,148	3	pXS806-1:21,750 bp; pXS806-2:26,800 bp; pXS806-3:21,750 bp	12	71	1991

**Table 2:** General overview of 21 retrieved *L. mesenteroides* genomes.

Strain	NCBI RefSeq Assembly	Level	Source	Size (Mb)	GC Content (%)	Scientific Name	References
DRC1506	GCF_001886915.1	Complete	kimchi	1.98	37.5	*L. mesenteroides*	NCBI RefSeq
WiKim0121	GCF_023809725.1	Complete	kimchi	2.19	37.5	*L. mesenteroides* subsp. *dextranicum*	NCBI RefSeq
WiKim32	GCF_019048965.1	Complete	kimchi	2.13	37.5	*L. mesenteroides* subsp. *mesenteroides*	NCBI RefSeq
MSL129	GCF_029953235.2	Complete	kimchi	2.08	37.5	*L. mesenteroides*	NCBI RefSeq
KNU-2	GCF_021253825.1	Complete	kimchi	2.10	38	*L. mesenteroides*	NCBI RefSeq
PL03	GCF_024800105.1	Complete	kimchi	2.06	37.5	*L. mesenteroides*	NCBI RefSeq
SRCM217106	GCF_030369975.1	Complete	kimchi	2.00	37.5	*L. mesenteroides*	NCBI RefSeq
WiKim33	GCF_003433375.1	Complete	kimchi	1.97	37.5	*L. mesenteroides* subsp. *jonggajibkimchii*	NCBI RefSeq
wikim19	GCF_018336555.1	Complete	kimchi	1.80	38	*L. mesenteroides* subsp. *mesenteroides*	NCBI RefSeq
BD3749	GCF_001583825.1	Complete	Other Fermented vegetable	1.99	38	*L. mesenteroides*	NCBI RefSeq
DRC0211	GCF_002009375.1	Complete	kimchi	2.12	37.5	*L. mesenteroides*	NCBI RefSeq
SRCM210905	GCF_025515375.1	Contig	kimchi	2.09	37.5	*L. mesenteroides*	NCBI RefSeq
CECT 9217	GCF_900290445.1	Contig	Fermented potato	2.02	37.5	*L. mesenteroides* subsp. *mesenteroides*	NCBI RefSeq
838	GCF_025395095.1	Contig	Parsnips	1.98	37.5	*L. mesenteroides* subsp. *mesenteroides*	NCBI RefSeq
KIBGE-IB22	GCF_005049065.1	Scaffold	Fermented cabbage	1.95	37.5	*L. mesenteroides*	NCBI RefSeq
NBRC 3349	GCF_030269065.1	Scaffold	Fermented string beans	1.92	37.5	*L. mesenteroides*	NCBI RefSeq
DMLM18	GCF_045063855.1	Complete	kimchi	2.10	37.5	*L. mesenteroides*	NCBI RefSeq
yan	GCF_041344845.1	Complete	Green Onion	2.23	37.5	*L. mesenteroides*	NCBI RefSeq
DMLM17	GCF_045183415.1	Complete	kimchi	2.00	37.5	*L. mesenteroides*	NCBI RefSeq
CBA3650	GCF_040516045.1	Complete	kimchi	2.00	37.5	*L. mesenteroides*	NCBI RefSeq
MSJK0064	GCF_032919725.1	Complete	Other Fermented vegetable	2.00	37.5	*L. mesenteroides*	NCBI RefSeq

**Table 3:** Characterization of bacteriocin-encoding genes in 29 *L. mesenteroides* strains.

Strain	Number of Areas of Interest (AOI’s)	Location	AOI ID	Bacteriocin Class
AP7	1	chromosome	Contig00001.2.AOI_01	Enterocin Xβ
C5	0			
XM4	1	chromosome	Contig00001.2.AOI_01	Enterocin Xβ
XM27	1	chromosome	Contig00001.1.AOI_01	Enterocin Xβ
XS14	1	chromosome	Contig00001.1.AOI_01	Enterocin Xβ
XS804	1	chromosome	Contig00001.0.AOI_01	Enterocin Xβ
XS806	1	chromosome	Contig00001.3.AOI_01	Enterocin Xβ
XM7	1	chromosome	Contig00001.1.AOI_01	Enterocin Xβ
DRC1506	1	chromosome	CP0146111.0.AOI_01	Enterocin Xβ
WiKim0121	1	chromosome	CP0987841.1.AOI_01	Enterocin Xβ
WiKim32	1	chromosome	CP0377521.4.AOI_01	Enterocin Xβ
MSL129	0			
KNU-2	1	chromosome	CP0897821.4.AOI_01	Enterocin Xβ
PL03	0			
SRCM217106	1	chromosome	CP1284961.3.AOI_01	Enterocin Xβ
WiKim33	1	chromosome	CP0214911.2.AOI_01	Enterocin Xβ
wikim19	1	chromosome	CP0480061.0.AOI_01	Enterocin Xβ
BD3749	1	chromosome	CP0146101.0.AOI_01	Enterocin Xβ
DRC0211	0			
SRCM210905	1	chromosome	JAOPIY0100000011.2.AOI_01	Enterocin Xβ
CECT 9217	1	contig	OKRL010000041.0.AOI_01	Enterocin Xβ
838	1	contig	QVOR010000021.3.AOI_01	Enterocin Xβ
KIBGE-IB22	1	Scaffold	SUNK010000051.8.AOI_01	Enterocin Xβ
NBRC 3349	1	Scaffold	BSSJ010000071.10.AOI_01	Enterocin Xβ
DMLM18	1	chromosome	CP1736871.1.AOI_01	Enterocin Xβ
yan	1	chromosome	CP1668261.4.AOI_01	Sonorensin
DMLM17	1	chromosome	CP1740031.2.AOI_01	Enterocin Xβ
CBA3650	1	chromosome	CP1594651.0.AOI_01	Enterocin Xβ
MSJK0064	1	chromosome	CP1366681.1.AOI_01	Enterocin Xβ

## Data Availability

All relevant data of this study are presented. Additional data will be provided upon request.

## Supplementary Materials

The following supporting information can be downloaded at: https://www.mdpi.com/article/10.3390/microorganisms14061350/s1, Supplymentary Table S1: Sequencing and assembly quality; Supplementary Table S2: Plasmid-gene prokka annotation; Supplymentary Table S3: Prophage prediction of the 29 genomes; Supplementary Table S4: Genomic island; Supplymentary Table S5: Bacteriocins; Supplementary Table S6: Antibiotic resistance.
